# Predicting extrusion process parameters in Nigeria cable manufacturing industry using artificial neural network

**DOI:** 10.1016/j.heliyon.2020.e04289

**Published:** 2020-07-28

**Authors:** Ayokunle Adesanya, Ademola Abdulkareem, Lambe Mutalub Adesina

**Affiliations:** a Department of Electrical and Information Engineering, Covenant University, Sango Ota, Ogun State, Nigeria; b Department of Electrical and Computer Engineering, Kwara State University, Kwara State, Nigeria

**Keywords:** Electrical engineering, Industrial engineering, Mechanical engineering, Computer-aided engineering, Electrical cable, Ann, Extrusion, Extruder

## Abstract

The extrusion process is a very complex process due to the number of process parameters that are associated with it which are prone to high fluctuations. The main purpose of this work is to determine the realistic extrusion process parameters in the thermoplastic extrusion process in Nigeria cable manufacturing industries with the use of an artificial neural network. Conventionally, the use of trial and error technique which involves full-size experiments is generally used to determine the process parameters in the thermoplastic extrusion process. This conventional technique is expensive and it is also time-consuming. The use of an artificial neural network to predict extrusion process parameters before plant execution will make extrusion process operations more efficient. This technique also bridges the gap that exists between theoretical analysis and real manufacturing system because real manufacturers' data was used. The neural network was developed in a MATLAB environment and was trained with a supervised learning method based on Levenberg Marquardt Algorithm and the developed ANN model is capable of predicting manufacturing process parameters for different grades of PVC thermoplastic material.

## 1 Introduction

The extrusion process is a manufacturing technique in which materials are moved along a screw and are pushed out through a die at a certain temperature and pressure. One of the most common places the extrusion process is utilized is in the cable manufacturing industries. It is very useful in the extrusion of thermoplastic material (PVC, PE, or XLPE) in electrical cable insulation [1, 2]. One of the most common thermoplastic PVC [3]. Cable manufacturing in Nigeria today is faced with some challenges which can affect the quality of the cables [4]. The challenges are often associated with the complex processes that are involved in the manufacturing process of the cables. In cable manufacturing industries, there are a vast amount of parameters (known as process parameters) that affect the output product obtained after the extrusion process [4, 5]. The process parameters include the melt temperature, speed, pressure settings, screw speed, type of die used and cooling medium in an extrusion machine. The melt temperature and pressure are some of the most important parameters in an extrusion process. These parameters indicate the performance quality of an extruder [6]. The melt temperature variations in an extruder are also important to determine the stability of the extrusion process [7]. Krzysztof stated that the temperature, pressure, and the fluctuations in the process parameters in an extruder are essential to determine the quality of an extrusion output. Other properties include the degree at which the polymer compositions are well mixed and the rheological properties of the polymer [8]. These process parameters are very prone to fluctuations, therefore cause issues in cable manufacturing industries [9]. Some of these issues include the production of poor quality cables, an increase in the manufacturing cost and time and as well as the waste of materials in manufacturing industries [10].

**Figure 1:**
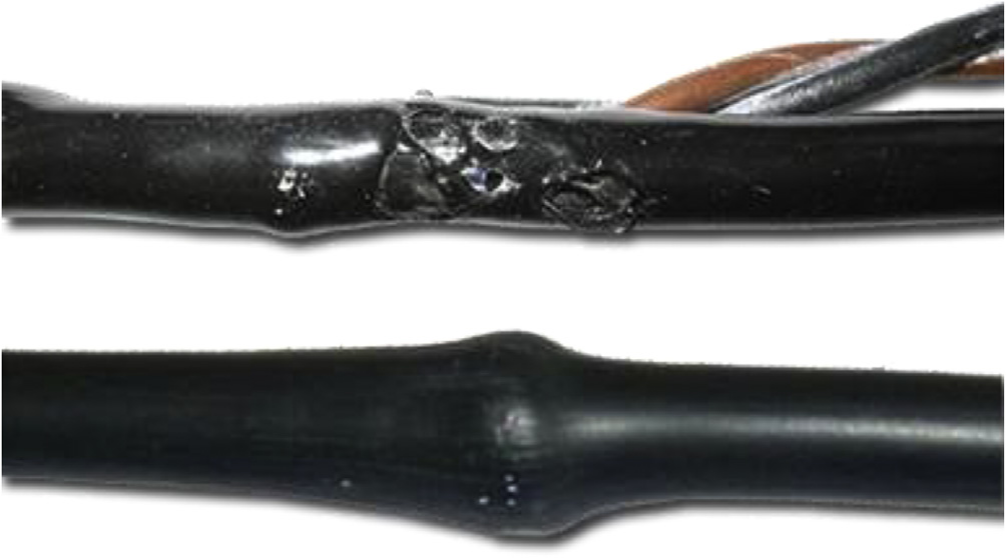
Electrical cable insulation with defects.

**Figure 2:**
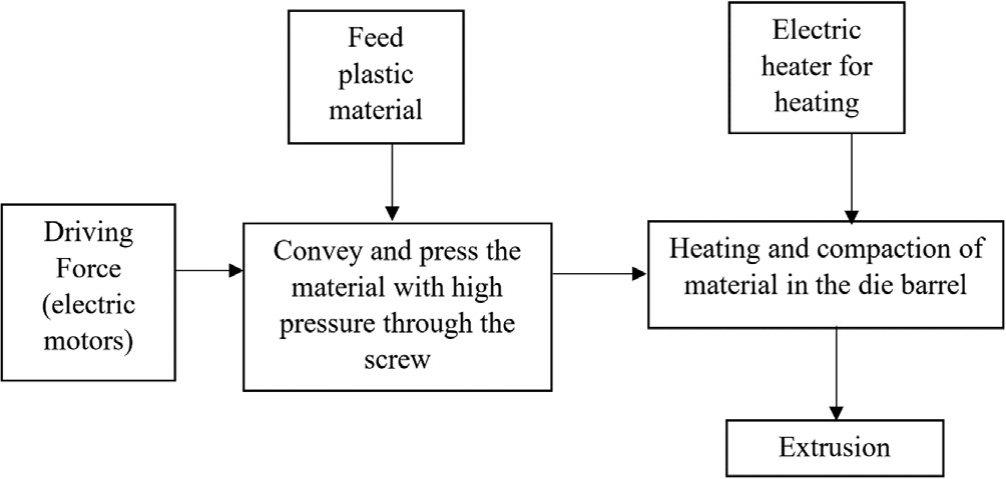
Block diagram of Extrusion process.

**Figure 3:**
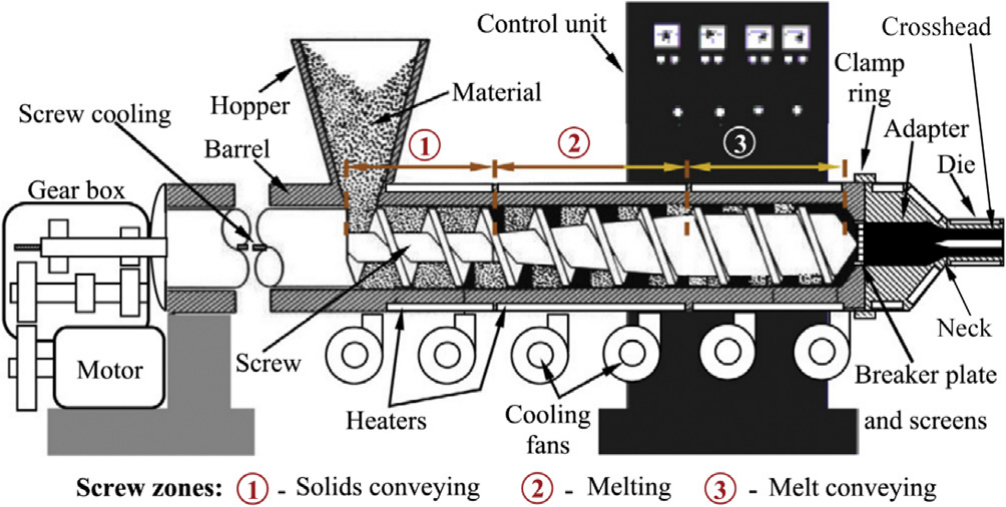
Schematic diagram of a single screw extruder.

The complexity and the high number of process parameters that are involved in the manufacturing of these cables make the entire process difficult to control and monitor [11]. Improper operations in the manufacturing process often cause defects in the cables manufactured [4]. These defects can be in the form of pimples, cracking, air cavity, and porosity in cable insulations [4]. Figure 1 shows the diagrammatic representation of an electrical cable with defects. Electrical cable insulation defects can lead to insulation breakdown that can lead to the loss of life and properties. The improper operations in electrical cable manufacturing industries can also lead to an increase in manufacturing cost, downtime, and waste of material [12]. Furthermore, it can also cause a reduction in economic benefits while increasing energy and labor [12, 13]. Therefore, discovering ways to improve the manufacturing process of electrical cables to provide quality outputs is very important. Figure 2 shows the block diagram of a typical extrusion process technique and Figure 3 shows the schematic diagram of an extruder.

Over the years, many methods have been developed to optimize the extrusion process in the industry. These techniques are developed to ensure that the production of high-quality extrusion output is achieved while reducing manufacturing cost, downtime, waste of material, energy, labor, and increasing economic benefits [9, 13]. Some researchers investigated how the thermoplastic extrusion process can be improved in the industry. They asserted that the quality of the PVC which is used in the cable industry can impact the output quality from an extruder [4]. Jing and colleagues proposed a low-cost real-time energy monitoring method which is used to study the effect of process settings on efficiency and melt quality [14, 15]. Chamil and colleagues also established that energy efficiency is a concern and they identified ways to optimize energy efficiency [16]. Zinnatullin and colleagues investigated the use of an automatic control system in the extrusion of polymer cable insulation [17]. Abeykoon proposed a model-based controller that can be utilized in a polymer extrusion process. In this work, the author stated that since the melt temperature is a major process parameter that can impact the output of an extruder, there is a need for accurate thermal monitoring and control [18]. The proposed system was capable of achieving the melt temperature that was desired and it also reduces the temperature variance in the extrusion line. Pathak and colleagues investigated the effects of the process parameters in the extrusion process by utilizing the finite element method [19]. The best process parameters for hot extrusion was also studied by Sivaprasad and colleagues by using finite element simulation [20, 21]. Dharmendra and Sunil proposed a method of optimizing the process parameters of high-density polyethylene (HDPE) material using the Taguchi approach [22, 23]. Many other researchers have utilized the Taguchi approach to obtain great results in extrusion processes [22, 23, 24]. Vicente and colleagues proposed the use of regression models to predict the quality in a tubing extrusion process. They discussed how quality cannot be overemphasized in the manufacturing process [25, 26]. Chamil and colleagues developed an extruder melt temperature control with fuzzy logic [27]. Nastaj and Wilczynski in their work were able to optimize the single screw extrusion process using process simulations [28]. The process simulation technique was based on Genetic Algorithms Screw Extrusion Optimization procedures which have been developed using the Genetic Algorithm. This technique was used to optimize both the starve and flood fed screw extrusion. The authors were able to conclude that the starving fed extrusion is optimal with a high extrusion output and low energy consumption [28]. Bingol and some of his colleagues developed an ANN model to predict the load for lateral extrusion. In their study, the authors used the billet diameters, height, and teeth for input parameters and the results shows that the ANN model developed was capable of estimating the required load for lateral extrusion [29]. Furthermore, Carmine and colleagues also developed an ANN model to optimize the extrusion of aluminum alloy [30]. In their study, they postulated that the extrusion of aluminum alloy is complex which is due to the materials and process parameters used in the extrusion process. The authors used Levenberg Marquardt algorithm to train an ANN model using experimental data from an industrial process. In their study, they predicted the temperature profile of an extruder machine using ANN and they were able to agree that the predicted values are very close to the experimental values [30]. Other researchers such as Su-Hai Hsiang et al. [31] and Mekras [32] have also utilized ANN in the extrusion process and the results were satisfactory. These have shown that the use of ANN in the extrusion process is not entirely new and the results that have been obtained in these processes are relevant to the reasons why the use of ANN has been utilized in the study. This study focuses on the use of artificial neural networks to predict extrusion process parameters in cable manufacturing industries. The method provides a better way of selecting process parameters that can be used in cable manufacturing industries. The prospects of using artificial neural network controllers in the PVC thermoplastic extrusion process were also discussed.

## 2 Methods and materials

### 2.1 Dataset material

In this study, the Polyvinyl Chloride (PVC) thermoplastic material was considered. The relevant data of the appropriate process parameters settings as well as the datasheet of different grades of PVC thermoplastic material was obtained from two cable manufacturing industries. The two cable manufacturing industry were selected based on their capability to produce high-quality cables and easy accessibility. Forty-two (42) different PVC grade material was utilized in this study. The datasheet of the PVC grade materials was obtained from two cable manufacturing industries. The datasheet information was used as the input parameters in the model. Furthermore, the industrial process parameters settings which are used in the extrusion process of these materials were also obtained from the cable manufacturing industries. These process parameters settings were used as the output parameters of the developed ANN model. The artificial neural network model was developed in a MATLAB environment. The datasheet that was obtained for the PVC thermoplastic extrusion can be seen in Tables 1, 2 and 3. The process parameters that were considered include the zone temperatures, clamp temperature, neck temperature, crosshead temperature, and the die temperature. Tables 1, 2, and 3 shows the training datasets while Table 4 shows the testing dataset that was utilized in this study.

**Table 1:** Dataset for different grades of PVC thermoplastic material (1).

Grade Name (input)	KI-041S	KI-09	KI-11	KI-12	KI-13K	KI-14	KI-15SC	KI-21	KI-22	KI-25J	KI-06S	KI-06	KI-05
Maximum Operating temperature (°C)	70	70	70	70	70	70	80	85	85	85	70	70	70
Specific Gravity (G/CC)	1.46	1.47	1.46	1.38	1.38	1.4	1.35	1.38	1.38	1.38	1.56	1.47	1.47
Hardness, (Shore A)	90	91	90	88	87	90	93	88	90	90	90	86	86
Thermal Stability (°C)	110	90	100	80	120	120	120	150	200	200	60	60	60
Tensile Strength, TS (MPa)	16	15	15	17	17	17	20	16	16	16	15	15	15
Elongation at break, EB (%)	250	250	250	300	275	250	280	250	250	250	200	250	250
Ageing Temperature (°C)	80	80	80	80	80	80	100	135	135	135	90	80	80
Variations of TS (%)	20	20	20	20	20	20	25	25	25	25	20	20	20
Variations of EB (%)	20	20	20	20	20	20	25	25	25	25	20	20	20

Profile Settings (target)													

1st Zone (°C)	130	131	130	129	129	132	125	130	132	132	160	131	131
2nd Zone (°C)	150	155	150	153	153	153	150	153	154	154	180	157	157
3rd Zone (°C)	160	165	160	160	160	162	163	160	160	160	185	160	160
4th Zone (°C)	160	160	165	159	159	160	164	160	160	160	185	160	160
5th Zone (°C)	165	160	165	161	161	162	165	160	160	160	185	160	160
6th Zone (°C)	160	162	160	161	160	161	160	160	160	160	185	162	162
7th Zone (°C)	160	165	160	160	171	160	160	160	160	160	185	165	165
Clamp (°C)	170	170	172	170	155	171	175	171	175	175	190	170	170
Neck (°C)	155	155	155	156	170	155	150	155	150	150	160	154	154
Crosshead (°C)	175	170	170	170	169	170	170	170	170	170	170	170	170
Die (°C)	180	170	180	175	175	175	175	175	175	175	165	170	170

**Table 2:** Dataset for different grades of PVC thermoplastic material (2).

Grade Name (input)	KI-75	KI-02	KI-26	KI-42	KI-31	KI-04FR	KI-33B	KI-26	KI-05	KI-25JW	KI-23	KI-05	KI-15
Maximum Operating temperature (°C)	90	90	90	90	90	70	90	90	86	95	93	86	93
Specific Gravity (G/CC)	1.44	1.48	1.47	1.47	1.5	1.5	1.57	1.48	1.47	1.33	1.38	1.47	1.38
Hardness, (Shore A)	80	90	90	88	90	90	92	90	86	95	93	86	93
Thermal Stability (°C)	80	120	120	100	120	90	150	60	60	240	100	60	100
Tensile Strength, TS (MPa)	16	15	15	15	15	15	14	14	13	16	17	13	17
Elongation at break, EB (%)	300	275	250	250	250	250	200	250	250	250	250	250	250
Ageing Temperature (°C)	80	100	100	100	100	90	100	80	80	135	80	80	80
Variations of TS (%)	20	20	20	20	20	20	20	20	20	25	25	20	25
Variations of EB (%)	20	20	20	20	20	20	20	20	20	25	25	20	25

Profile Settings (target)													

1st Zone (°C)	135	130	130	130	158	158	160	130	130	120	130	130	130
2nd Zone (°C)	155	150	150	150	180	180	180	145	150	135	155	150	155
3rd Zone (°C)	160	165	165	165	180	180	185	150	165	140	160	165	160
4th Zone (°C)	160	160	160	160	180	180	185	160	160	150	160	160	160
5th Zone (°C)	162	160	160	160	180	180	185	160	160	150	160	160	160
6th Zone (°C)	165	162	160	160	180	180	185	165	160	155	160	160	160
7th Zone (°C)	165	165	165	170	180	180	185	165	175	155	165	175	165
Clamp (°C)	170	170	170	170	185	185	190	170	175	160	175	175	175
Neck (°C)	155	155	155	155	160	160	160	155	155	160	150	155	150
Crosshead (°C)	170	170	170	170	165	165	170	170	170	160	170	170	170
Die (°C)	170	170	170	170	150	150	165	170	170	155	175	170	175

**Table 3:** Dataset for different grades of PVC thermoplastic material (3).

Grade Name (input)	KI-06	ASTM 668-96	ASTM A975-97	KI-06H	KI-10H	KI-26H	KI-AVSS	KI-10	KI-09H	KI-AV
Maximum Operating temperature (°C)	92	93	96	70	70	90	70	70	105	70
Specific Gravity (G/CC)	1.45	1.4	1.34	1.47	1.38	1.47	1.34	1.36	1.35	1.37
Hardness, (Shore A)	92	93	96	86	88	88	90	90	92	90
Thermal Stability (°C)	80	100	100	60	80	100	100	100	110	90
Tensile Strength, TS (MPa)	15	15	15	15	17	15	15	15	17	15
Elongation at break, EB (%)	250	250	250	250	300	250	180	180	250	180
Ageing Temperature (°C)	80	63	63	80	80	100	100	100	135	100
Variations of TS (%)	20	20	25	20	20	20	90	90	25	90
Variations of EB (%)	20	20	25	20	20	20	70	70	25	70

Profile Settings (target)										

1st Zone (°C)	128	132	131	131	129	130	120	130	130	130
2nd Zone (°C)	145	155	155	157	153	150	140	154	154	153
3rd Zone (°C)	155	161	162	160	160	165	140	160	160	160
4th Zone (°C)	160	159	161	160	159	160	150	160	160	159
5th Zone (°C)	160	161	160	160	161	160	150	160	165	161
6th Zone (°C)	160	160	161	162	161	160	155	160	165	160
7th Zone (°C)	175	161	160	165	160	170	155	165	165	171
Clamp (°C)	175	171	171	170	170	170	165	175	175	155
Neck (°C)	155	154	155	154	156	155	165	150	150	170
Crosshead (°C)	165	171	170	170	170	170	160	170	170	169
Die (°C)	170	169	169	170	175	170	155	175	175	175

**Table 4:** Testing Dataset for different grades of PVC thermoplastic material.

Grade Name (predict)	KI-16H	KI-AV10	KI-26H	KI-AV2	ASTM 667-98	ASTM 668-98
Maximum Operating temperature (°C)	70	90	70	70	80	85
Specific Gravity (G/CC)	1.38	1.47	1.4	1.38	1.35	1.38
Hardness, (Shore A)	88	88	90	87	93	90
Thermal Stability (°C)	80	100	120	120	120	200
Tensile Strength, TS (MPa)	17	15	17	17	20	16
Elongation at break, EB (%)	300	250	250	275	280	250
Ageing Temperature (°C)	80	100	80	80	100	135
Variations of TS (%)	20	20	20	20	25	25
Variations of EB (%)	20	20	20	20	25	25

Profile Settings (Expected Values)						

1st Zone (°C)	129	130	132	129	125	132
2nd Zone (°C)	153	150	153	153	150	154
3rd Zone (°C)	160	165	162	160	163	160
4th Zone (°C)	159	160	160	159	164	160
5th Zone (°C)	161	160	162	161	165	160
6th Zone (°C)	161	160	161	160	160	160
7th Zone (°C)	160	170	160	171	160	160
Clamp (°C)	170	170	171	155	175	175
Neck (°C)	156	155	155	170	150	150
Crosshead (°C)	170	170	170	169	170	170
Die (°C)	175	170	175	175	175	175

### 2.2 Artificial neural network

**Figure 4:**
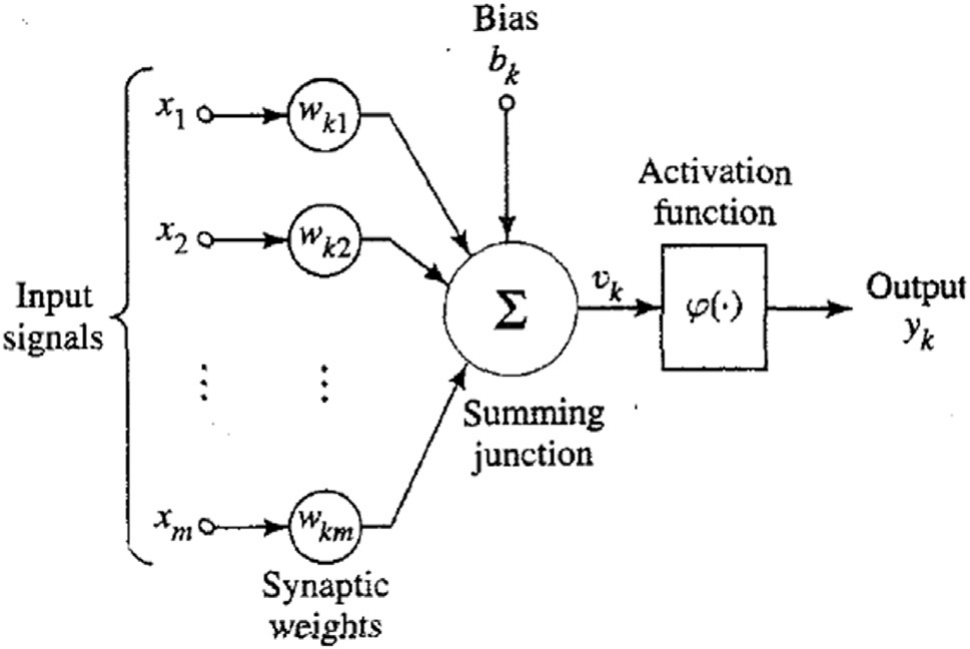
Diagrammatic representation of a neuron.

Artificial Neural Network, popularly known as ANN is a machine learning technique/algorithm which is inspired by the biological nervous system. ANN is a machine learning technique that is capable of exploring the relationships between different variables with very high accuracy. Artificial neural network emulates the human neurological system to be able to analyze and discover patterns from historical data. ANN models the way a brain performs a particular task or function. ANN due to its computing power can learn and generalize. Generalization is the ability of neural networks to produce reasonable outputs for inputs that are not used during training. ANN is composed of processing units known as neurons which is an information processing unit that is fundamental to the operation of a neural network. Figure 4 shows the model of a neuron.

A neuron consists of connecting links with weights, an adder, and an activation function for limiting the output of a neuron (squashes the amplitude range of the output signal to a finite value).

Mathematically, a neuron can be described by the following equations:

(1) $$v_{k}=\sum_{j=0}^{m}w_{kj}x_{j}+b_{k}$$

(2) $$y_{k}=\phi \left(v_{k}\right)$$

$x_{1},x_{2},x_{3}\ldots x_{m}$ are the input signals; $w_{k1},w_{k2},\ldots w_{km}$ are the synaptic weights of neuron k. $v_{k}$ is the linear combiner output due to the input signal; $b_{k}$ is the bias; ϕ(.) is the activation function; and $y_{k}$ is the output signal. Different types of activation functions can be used in an ANN model. Some common types include the sigmoid, linear, Gaussian, and gaussian complement functions. However, the most commonly used type is the sigmoid function which was also used in this study. The sigmoid function can be expressed mathematically in Eq. (3). Artificial neural networks also consist of different types of models which include the multilayer perceptron (MLP), wavelet neural network, Elman neural network, radial basis, etc. In this work, the multilayer perceptron model was utilized in predicting the insulation thickness in the thermoplastic extrusion process.

(3) $$\phi \left(v_{k}\right)=\frac{1}{1+e^{-v_{k}}}$$

**Figure 5:**
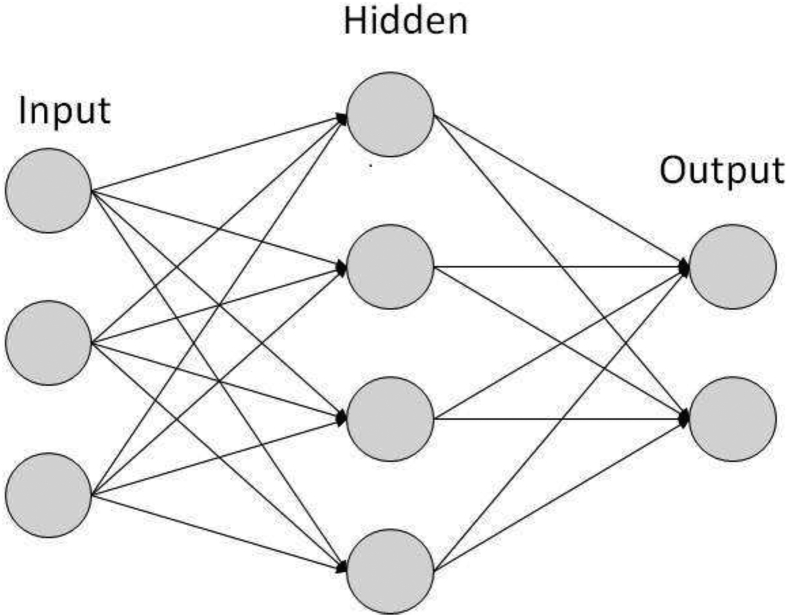
Architecture of an artificial neural network.

The use of ANN has been studied intensively since the 1990s. An ANN is capable of performing non-linear curve fitting and it is very suitable to predict the performance of the extrusion process as it is a non-linear process. The artificial neural network is inspired by the biological system of the human brain in the way it processes information. There are neurons in the human brain which are interconnected and are vital for receiving information through the connections. The ANN simply attempts to simulate the way the real neurons in the human brain behaves. The ANN is capable of learning by example the same way the human brain learns in real life. Depending on the type of problem, ANN can be applied in numerous ways. Figure 5 shows the interconnections that can be seen in an ANN.

Figure 5 shows an example of a feed-forward topology. The feed-forward topology is simply an architecture in which the signals at the input are moved in one way from the input to the output via the hidden layer. This can kind of topology can easily map an input to the output in a very quick and easy way. The feedback topology is another type of artificial neural network architecture. They differ from the feedforward topology because they can send signals in both directions in a network. These kinds of network topologies are very dynamic and they always change up until when equilibrium is accomplished. They are also quite complicated but are also very powerful. Other types of learning methods include the semi-supervised and the reinforcement learning algorithm.

### 2.3 Artificial neural network learning regime

In artificial neural networks, there are two distinct types of learning regimes. These are supervised and unsupervised learning. Supervised learning is a learning technique in which the output units are given the expected results to an input signal. The major problem with this type of learning regime are issues associated with convergence errors. The goal of the supervised learning regime is to reduce the error between a desired and computed value. Hence, appropriate weighting functions are developed to minimize these errors. A particular type of training known as backpropagation is a type of supervised learning regime and it is most commonly used during the training of an artificial neural network. In the backpropagation technique, a training sample is fed into the network and the appropriate output values are obtained based on the provided inputs in the forward direction. The errors obtained at the output neurons are then propagated back to the input layer where the weights are adjusted for each of the neurons and an appropriate algorithm is implemented to determine the weighting function that can reduce the error. The unsupervised learning regime, however, is when information is provided to the neural network with no desired target. The weight of the neurons is generally altered based on the type of response that is gotten from the input signals. When a set of information is provided, the artificial neural network randomly treats the information, and uses this information to obtain certain properties and produce the expected result. The supervised learning algorithm that was utilized in this study is the Levenberg Marquardt algorithm. The algorithm was able to appropriately produce accurate results with very high speed.

### 2.4 Multilayer perceptron

The multilayer perceptron is the building block for all neural network models. It consists of one input layer, one output layer, and one or more hidden layers. The input layer is responsible for receiving input data from an external source while the hidden layer receives inputs and sends the appropriate output in the network. The neurons of each of the layers are connected to the neurons of the following layer however, neurons on the same layer are not connected. The multilayer perceptron neural network developed in this work consists of one (1) input layer, two (2) hidden layers, and one (1) output layer.

### 2.5 Structure of the artificial neural network

**Figure 6:**
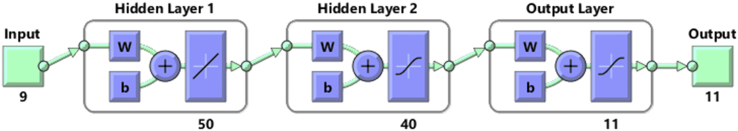
Schematic diagram of the neural network model.

**Figure 7:**
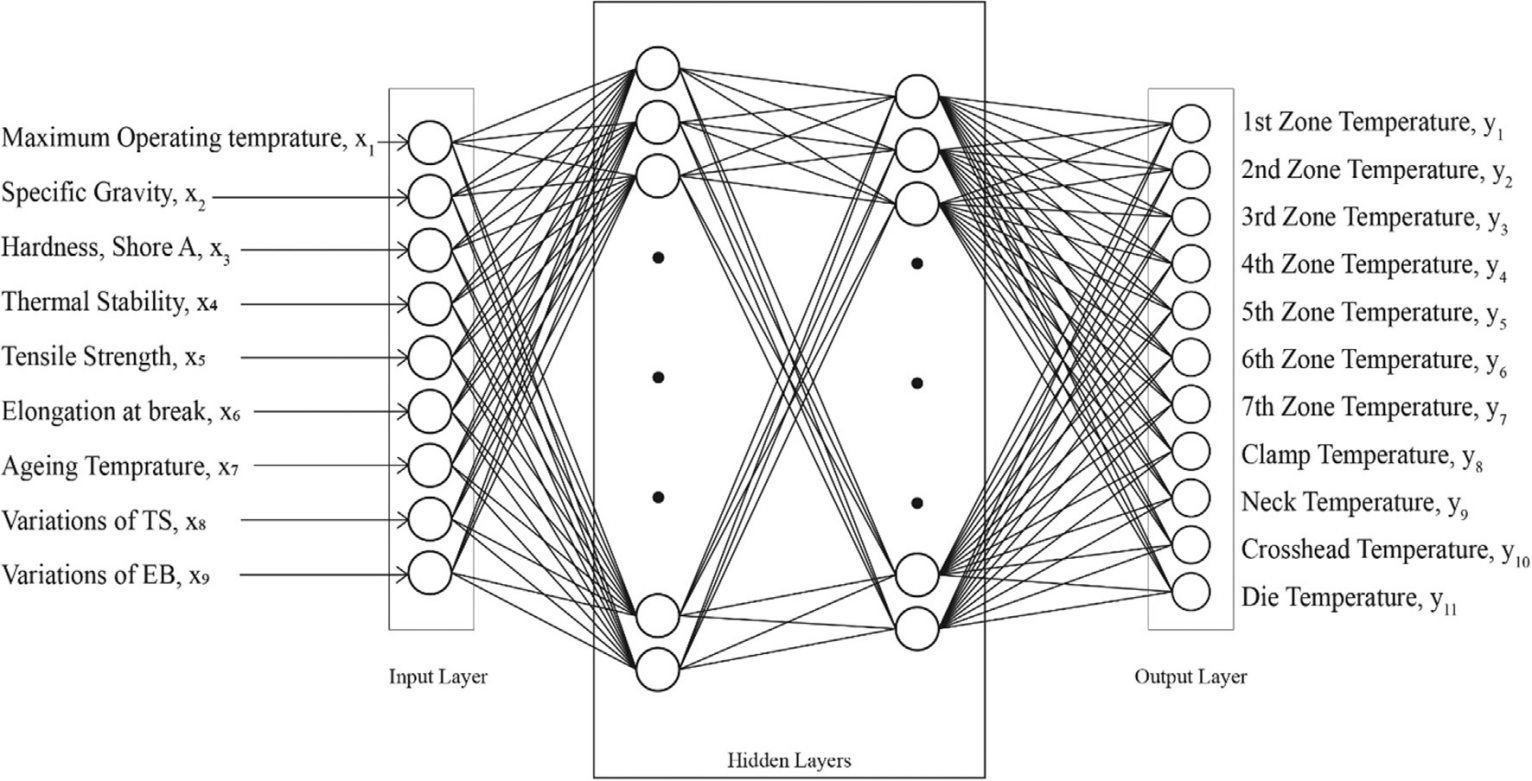
Network diagram for the neural network model for PVC extrusion process parameters prediction.

**Figure 8:**
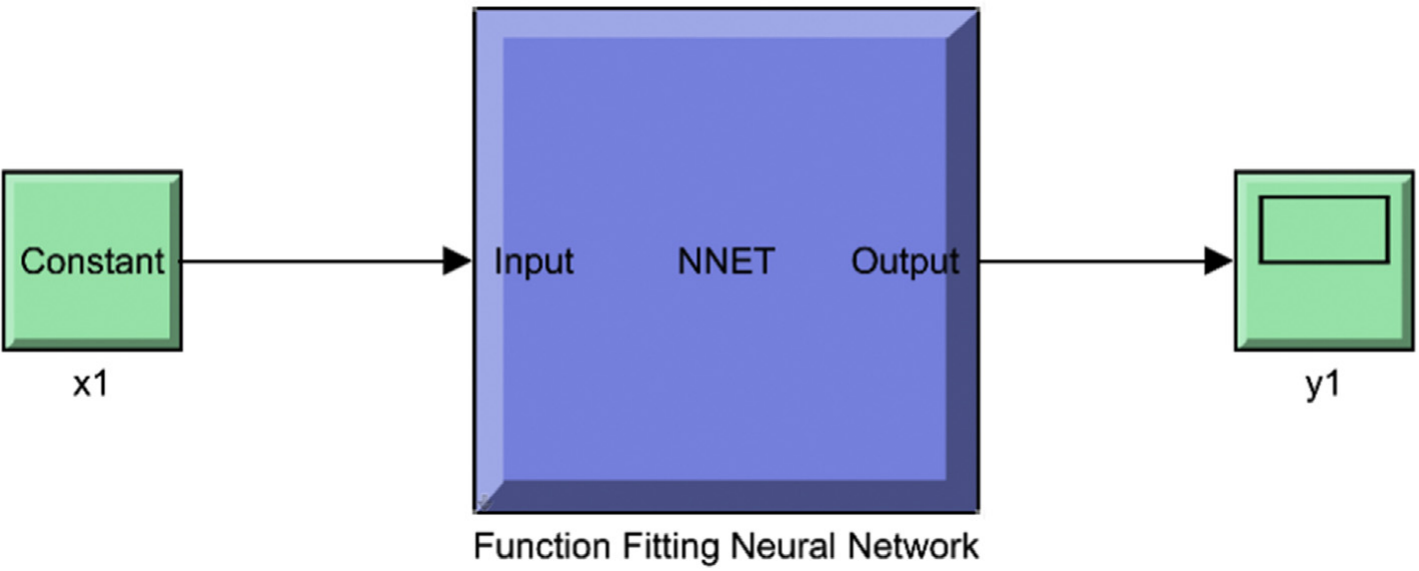
Function fitting neural network.

In this study, the ANN model was developed in MATLAB environment. Figure 6 shows the schematic diagram of the neural network model. The neural network consists of one input layer with nine (9) input neurons, two hidden layers with fifty (50), and forty (40) hidden neurons respectively, one output layer with eleven (11) neurons. The input parameters consist of the physical properties as obtained from the PVC material datasheet. These parameters are the maximum operation temperature, specific gravity, shore hardness, thermal stability, tensile strength, elongation at break, aging temperature, variations of the tensile strength (TS), and the variation of elongation break (EB). The output parameters of the model consist of the zonal temperature of the extruder machine from the first zone to the seventh zone, clamp, neck, crosshead, and die temperatures. The training algorithm that was utilized in this study was the Levenberg Marquardt algorithm and it provides the best result for the prediction of extrusion process parameters in PVC thermoplastic extrusion. Figures 7 and 8 shows the network diagram and the function fitting neural network for the proposed ANN model.

**Figure 9:**
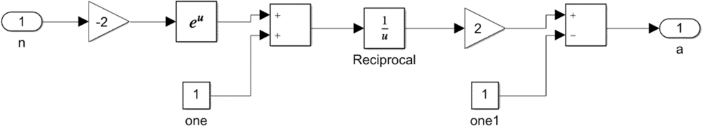
Tansig activation function Simulink diagram.

**Figure 10:**
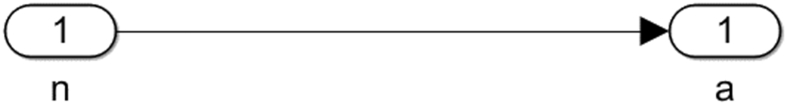
Purelin function Simulink diagram.

The activation function between the input and hidden layer is the Tansig function while the activation function between the hidden layer and the output layer is the Purelin function. Figures 9 and 10 represents the block representations of the Tansig and Purelin functions respectively.

Based on the network architecture utilized in this study, the mapping has two forms between the output and the input (independent) variables. The mapping is expressed in the equations below:

(4) $$Hidden\;Layer\to n_{k}^{\left(1\right)}=\sum_{n=1}^{R}w_{kn}^{\left(1\right)}x_{n}+b_{k}^{\left(1\right)}$$

(5) $$a_{k}^{\left(1\right)}=f_{1Level}(n_{k}^{\left(1\right)})$$

(6) $$Output\;Layer\to n_{k}^{\left(2\right)}=\sum_{n=1}^{R}w_{kn}^{\left(2,1\right)}a_{k}^{\left(1\right)}+b_{1}^{\left(2\right)}$$

(7) $$y=a_{k}^{\left(2\right)}=f_{2Level}\left(n_{k}^{\left(2\right)}\right)$$

where $w_{kn}$ are the weights of the links between the input layer and the hidden layer which are specific to independent variable n and neuron k, $b_{k}$ are the biases, $x_{n}$ are the input dataset, f is the activation function and y is the output. After successive iterations, the output equation can be generalized as follows [15]:

(8) $$y_{*}=f_{*}\left(\sum_{k=1}^{N}w_{k}^{'}f\left(\sum_{n=1}^{R}w_{kn}x_{n}+b_{k}^{\left(1\right)}\right)+b^{\left(2\right)}\right);n=1,2\ldots R\;k=1,2\ldots ,N$$

### 2.6 The Levenberg Marquardt Algorithm

One of the most efficient training algorithm used in ANN is the Levenberg Marquardt Algorithm. When we consider a neuron j with input p of a network with a $y_{j,i}$ number of neurons, and weights $w_{j,i}^{h}$, the output $y_{j}$is [33]:

(9) $$net_{j}^{h}=\sum_{i=1}^{n}\left(w_{j,i}^{h}y_{j,i}+\;b_{j}^{h}\right)\;$$

(10) $$y_{j}=f_{j}\left(net_{j}^{h}\right)$$

where h, $b_{j}^{h}$, $f_{j}$, and $net_{j}^{h}$ are the index, bias, activation function, and the sum of weighted input respectively. λ is a damping factor often used in the Levenberg Marquardt algorithm. The damping factor is often adjusted at every iteration until when the sum of the squared errors decreases. Eq. (11) shows the equation for the learning process.

(11) $$w_{k+1}=w_{k}-{\left(J_{k}^{T}J_{k}+\lambda I\right)}^{-1}J_{k}e_{k}$$

where w are the weights, J is the Jacobian matrix, and Je is the error gradient. To summarize, the Levenberg Marquardt algorithm can be summarized thus; the network weights are initialized and the sum square error (S) is calculated and evaluated. The Jacobian matrix is also computed and the error gradient is computed as well. The cross-product $J_{k}^{T}J_{k}$ is calculated and the equation $\left(J_{k}^{T}J_{k}+\lambda I\right)\Delta =Je$ is evaluated to find Δ. The network parameters are adjusted using Δ and the sum square errors are recalculated using the updated network's parameters. When the mean square error increase, the weights are changed to a former value and the damping factor is stepped up and the algorithm is done again. When the mean square error value decreases, the damping factor is reduced. The whole process is repeated with new weights value until the mean square error gets to the desired value.

### 2.7 Performance evaluation criteria

To be able to validate and evaluate the performance of the neural network developed, the mean square error (MSE) technique was utilized in this study. The values of the performance criteria must be as close to zero (0) as possible to indicate the high quality of the neural network developed. The performance criteria are described with the equations below:

(12) $$MSE=\;\frac{1}{n_{s}}\sum_{i=1}^{n_{s}}{\left(d_{i}-y_{i}\right)}^{2}$$

where $n_{s}$ is the number of observations, $d_{i}$ is the desired values and $y_{i}$ is the predicted value.

## 3 Results and discussions

### 3.1 Prediction extrusion process parameters

The results that were obtained from the prediction of the extrusion process parameters for PVC thermoplastic are discussed in this section. Each of the results for different grades of PVC thermoplastic material is presented with accompanying figures, tables, and graphical representations. The discussions and the relevance of the work were also clearly highlighted.

### 3.2 Prediction extrusion process parameters for PVC thermoplastic materials

A multilayer perceptron model (MLP) was developed to predict the extrusion process parameters for PVC thermoplastic material. The MLP developed consists of four layers. The number of layers and the accompanying number of neurons were determined by using a heuristic approach until the best result was obtained. The dataset consists of forty-two (42) different grades of PVC thermoplastic material. Each of these grades was accompanied by their corresponding properties and the appropriate profile settings from cable manufacturing industries. The input layer consists of nine (9) neurons with each neuron representing the property of the PVC material. These represent the input variables in the artificial neural network system. The two hidden layers consist of fifty (50) and forty (40) neurons respectively while the output layer consists of eleven (11) neurons which are the melt temperature profile settings. The melt temperature profile settings are the process parameters that represent the output variables. About fifteen percent (15%) of the entire dataset was used to test and validate the result of the neural network that was developed. This invariably means that bout six (6) different grades of PVC thermoplastic material which were not used in the training process were used to test the model to determine the accuracy of the system. Table 5 shows a summary of the MLP neural network design approach.

**Table 5:** Summary of the MLP neural network design approach.

Thermoplastic Material	PVC
Number of datasets	42
Training dataset	36
Validation dataset	6
Training Algorithm	Levenberg Marquardt
Activation Function	Tansig and Purelin
Training Time	5 s
Number of Iterations	6 iterations
Performance Evaluation	Mean Square Error
Number of Inputs Layers	1
Number of Input Neurons	9 (represents the properties for different grades of PVC thermoplastic)
Number of Hidden Layers	2
Number of Hidden Neurons (1^st^ layer)	50
Number of Hidden Neurons (2^nd^ layer)	40
Number of Output Layer	1
Number of Output Neuron	11 (represents output variable, i.e. profile settings)

**Figure 11:**
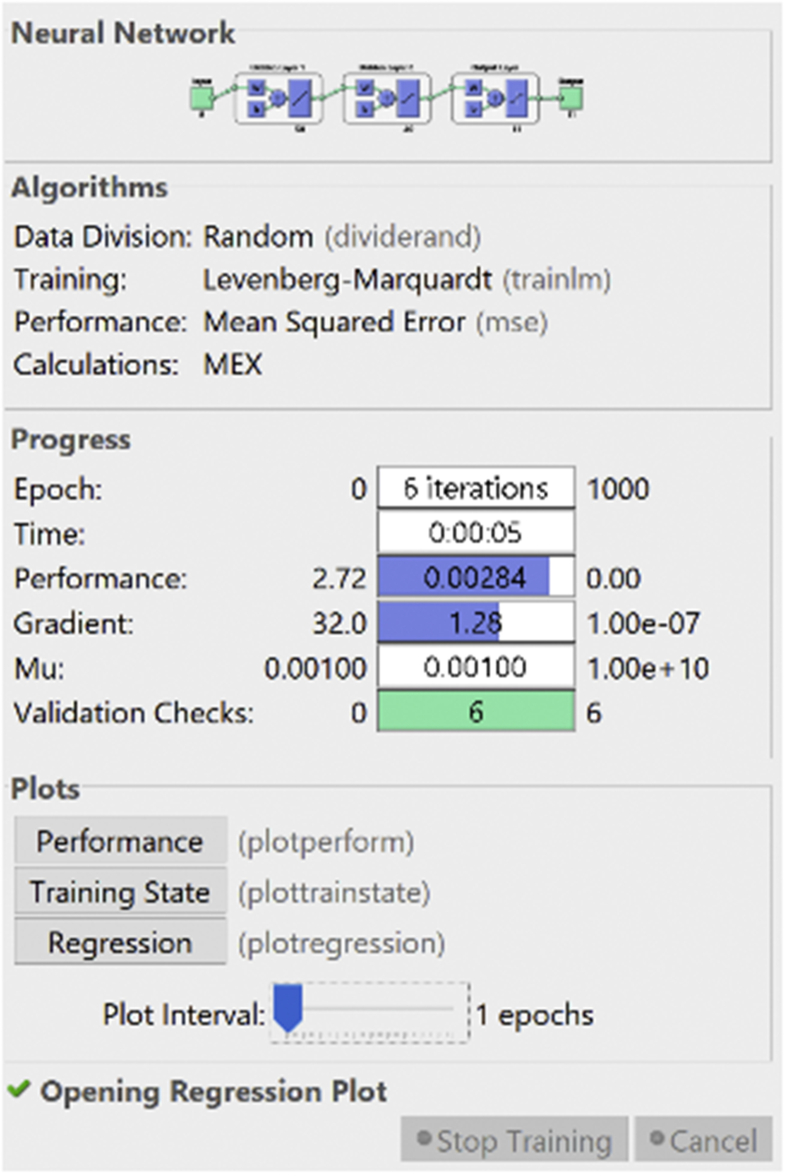
Training process result from the simulation in MATLAB environment (PVC process parameters).

**Figure 12:**
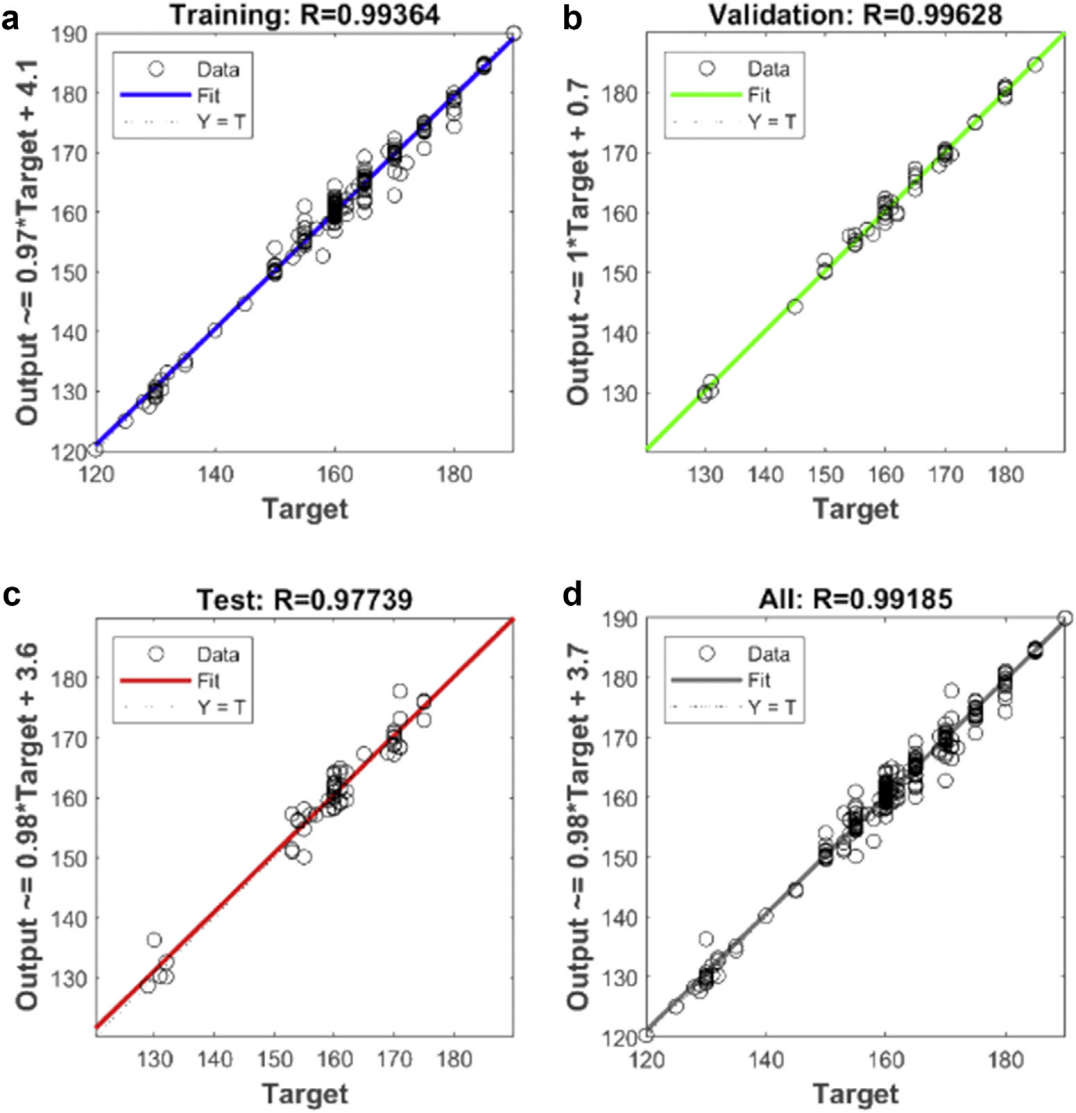
Regression analysis plot for Levenberg Marquardt algorithm (PVC process parameters). (a) – Training plot, (b) – Validation plot, (c) – Test plot, (d) – All plot.

The output results from the simulation in MATLAB can be seen in Figure 11. The performance, gradient as well as the training time of the developed neural network model can be observed from Figure 11. The regression plot for the Levenberg Marquardt neural network developed for the prediction of extrusion process parameters is shown in Figure 12. Table 6 shows the MSE and R values for the training, testing, and validation of the neural network.

**Table 6:** MSE and Regression values for the training, validation, and testing.

	MSE	R
Training	$3.85525\;\times 10^{-10}$	0.99364
Validation	$3.85525\;\times 10^{-6}$	0.99628
Test	$8.496995\;\times 10^{-6}$	0.97739
All	-	0.99185

**Figure 13:**
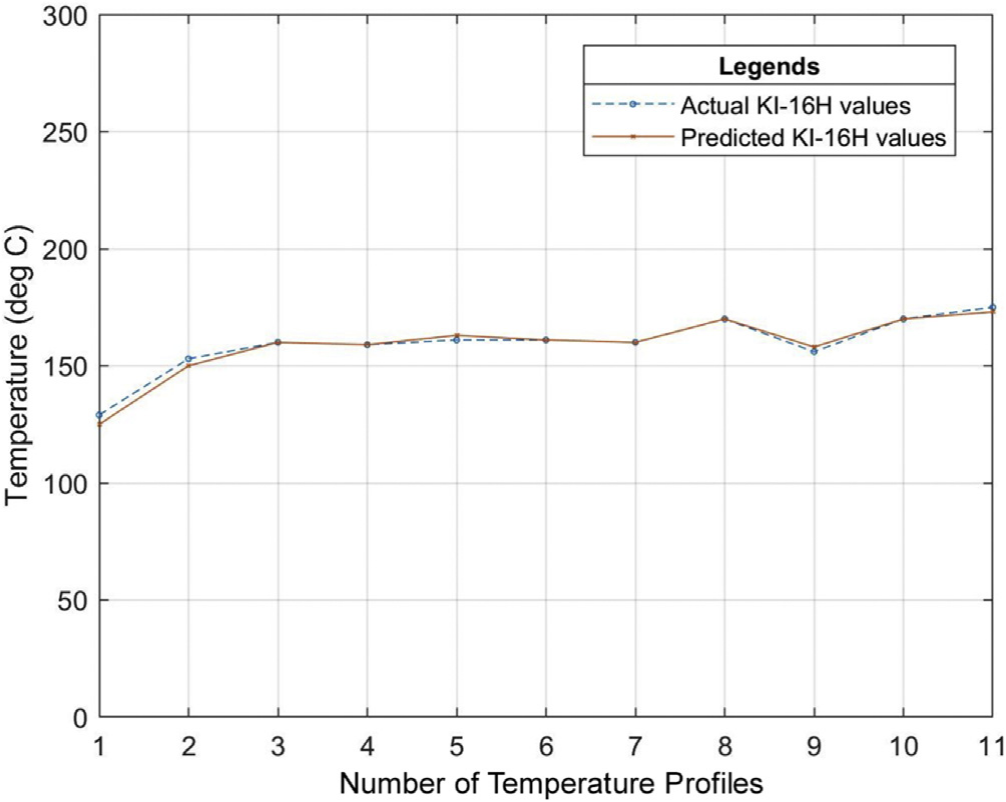
Relationship between actual values and production values (KI–16H).

**Figure 14:**
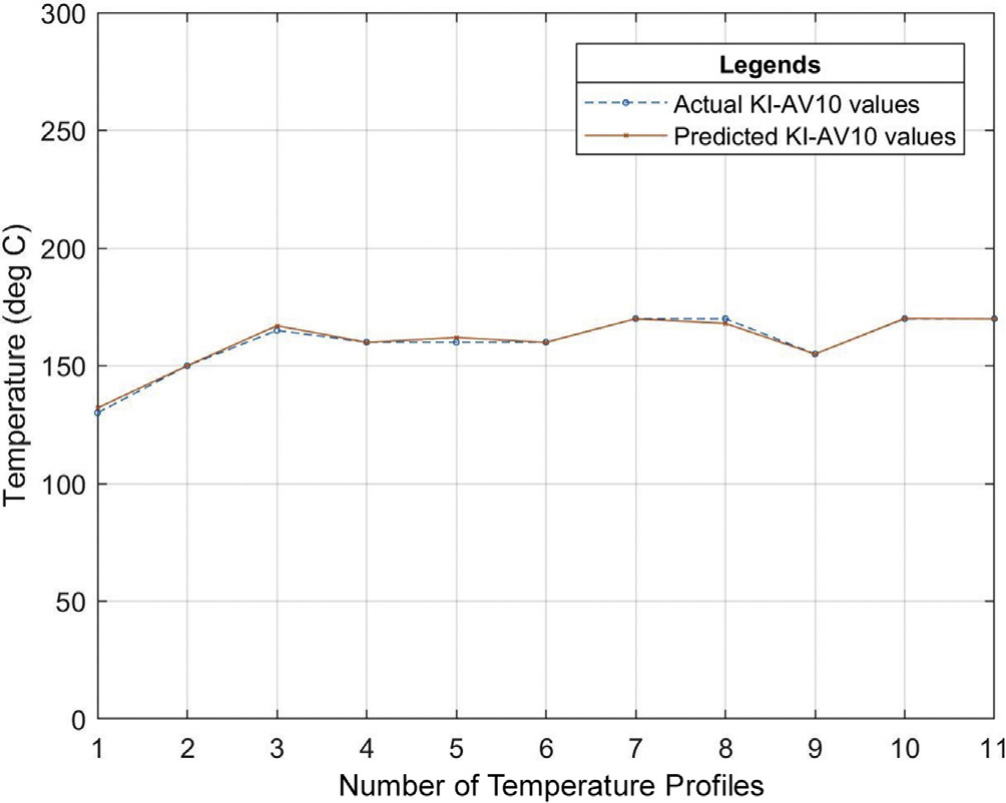
Relationship between actual values and production values (KI-AV10).

**Figure 15:**
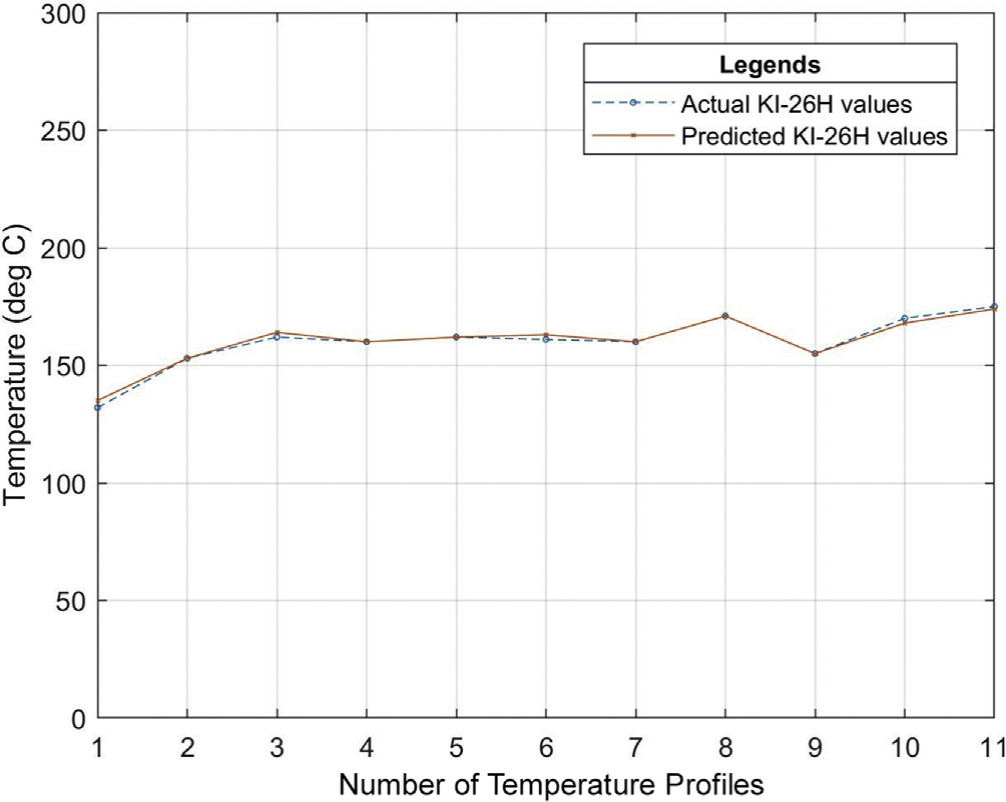
Relationship between actual values and production values (KI–26H).

**Figure 16:**
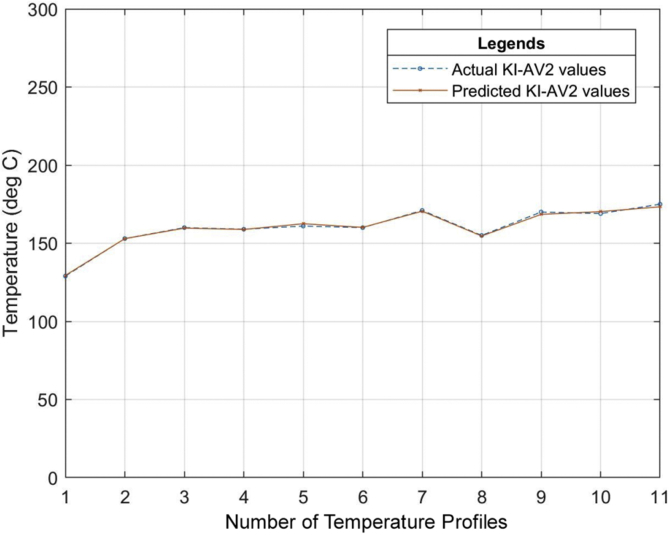
Relationship between actual values and production values (KI-AV2).

**Figure 17:**
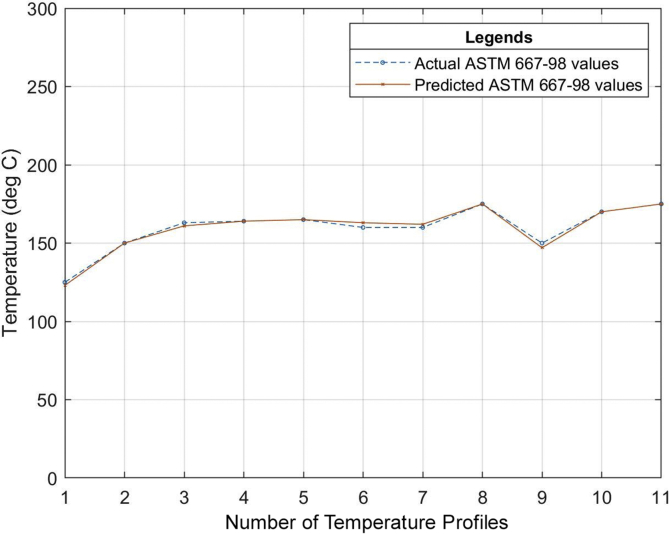
Relationship between actual values and production values (ASTM 667-98).

**Figure 18:**
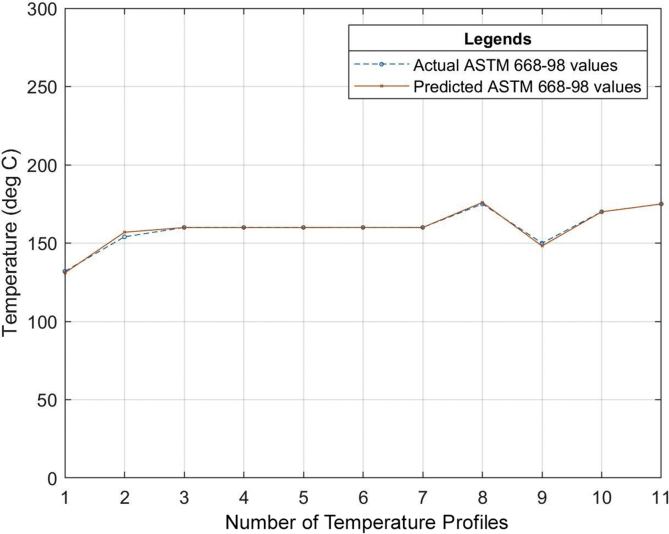
Relationship between actual values and production values (ASTM 668-98).

**Figure 19:**
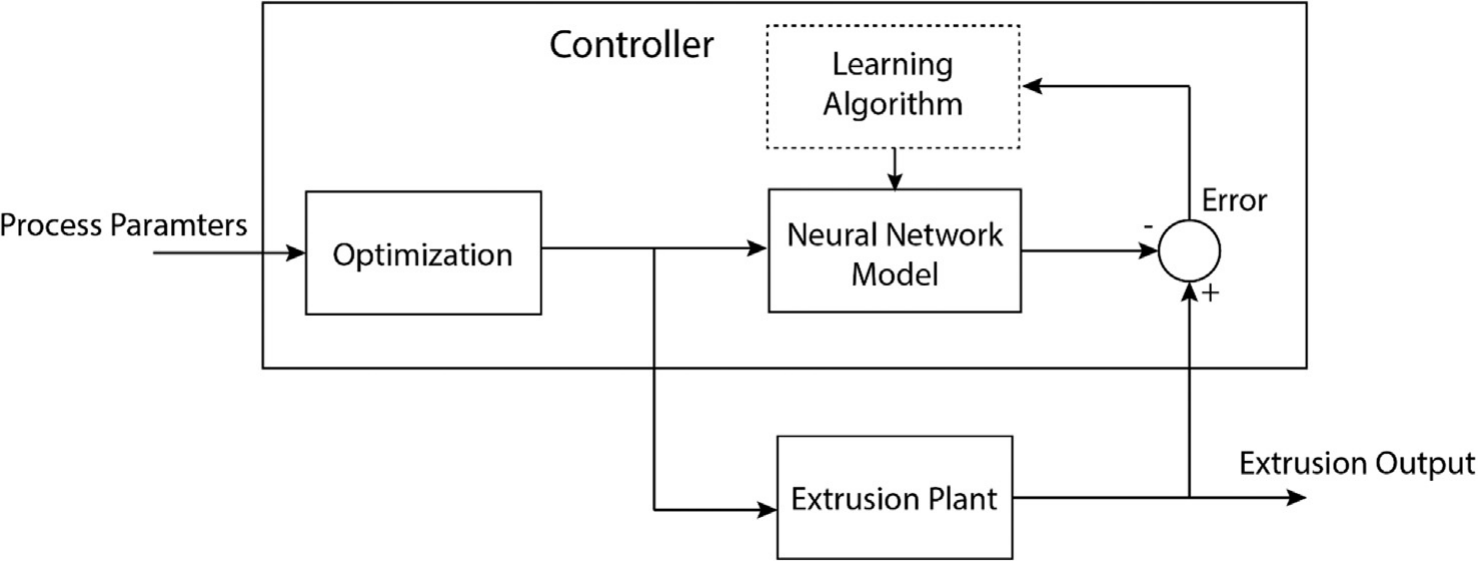
Block diagram of a proposed neural network controller in extrusion process.

Six (6) different grades of PVC material were used to test the developed neural network system. The different properties of the PVC materials such as the specific gravity, hardness, thermal stability, tensile strength, etc. all obtained from the datasheet of the PVC material as specified by the manufacturer were used as the input of the neural network that was developed. The relationship between the actual values for the process parameters settings (existing industrial values of the temperature profile) and the predicted values (temperature profile output of the ANN model) for the different grades of PVC are outlined in Tables 7, 8 and 9. The graphical representations of these relationships can also be seen in Figures 13, 14, 15, 16, 17, 18, and 19.

**Table 7:** Relationship between actual and predicted values for KI-16H and KI-AV10.

Name	KI-16H			KI-AV10		
Settings (°C)	Actual Value (°C)	Predicted Value (°C)	Error	Actual Value (°C)	Predicted Value (°C)	Error
1^st^ Zone	129	125.0032	3.9968	130	132.0817	-2.0817
2^nd^ Zone	153	150.0025	2.9975	150	150.0300	-0.03
3^rd^ Zone	160	159.9970	0.003	165	167.0148	-2.0148
4^th^ Zone	159	158.9953	0.0047	160	159.9504	0.0496
5^th^ Zone	161	162.9919	-1.9919	160	161.9800	-1.98
6^th^ Zone	161	161.0064	-0.0064	160	159.9505	0.0495
7^th^ Zone	160	159.9877	0.0123	170	169.9878	0.0122
Clamp	170	170.0038	-0.0038	170	168.0015	1.9985
Neck	156	157.9894	-1.9894	155	154.9835	0.0165
Crosshead	170	169.9988	0.0012	170	170.1180	-0.118
Die	175	173.0034	1.9966	170	169.9742	0.0258

**Table 8:** Relationship between actual and predicted values for KI-26H and KI-AV2.

Name	KI-26H			KI-AV2		
Settings (°C)	Actual Value (°C)	Predicted Value (°C)	Error	Actual Value (°C)	Predicted Value (°C)	Error
1^st^ Zone	132	135.0287	-3.0287	129	129.4109	-0.4109
2^nd^ Zone	153	153.0101	-0.0101	153	152.9500	0.05
3^rd^ Zone	162	164.0193	-2.0193	160	159.6972	0.3028
4^th^ Zone	160	160.0020	-0.002	159	158.8450	0.155
5^th^ Zone	162	162.0000	0	161	162.4825	-1.4825
6^th^ Zone	161	162.9937	-1.9937	160	160.1620	-0.162
7^th^ Zone	160	160.0191	-0.0191	171	170.5228	0.4772
Clamp	171	170.9839	0.0161	155	154.6700	0.33
Neck	155	155.0033	-0.0033	170	168.4966	1.5034
Crosshead	170	167.9737	2.0263	169	170.2469	-1.2469
Die	175	173.9614	1.0386	175	173.3428	1.6572

**Table 9:** Relationship between actual and predicted values for ASTM 667-98 and ASTM 668-98.

Name	ASTM 667-98			ASTM 668-98		
Settings (°C)	Actual Value (°C)	Predicted Value (°C)	Error	Actual Value (°C)	Predicted Value (°C)	Error
1^st^ Zone	125	123.0092	1.9908	132	130.9756	1.0244
2^nd^ Zone	150	150.0148	-0.0148	154	156.9521	-2.9521
3^rd^ Zone	163	160.9945	2.0055	160	159.9292	0.0708
4^th^ Zone	164	164.0042	-0.0042	160	159.9243	0.0757
5^th^ Zone	165	164.9903	0.0097	160	159.9500	0.05
6^th^ Zone	160	163.0020	-3.002	160	159.9783	0.0217
7^th^ Zone	160	162.0140	-2.014	160	159.9548	0.0452
Clamp	175	174.9839	0.0161	175	175.9558	-0.9558
Neck	150	147.0494	2.9506	150	148.1633	1.8367
Crosshead	170	169.9934	0.0066	170	169.9781	0.0219
Die	175	175.0047	-0.0047	175	174.9769	0.0231

The mean square error (MSE) for the PVC grades to determine the accuracy of the proposed model is as shown in Table 10.

**Table 10:** Mean Square Error obtained from the prediction of the extrusion process parameters of different PVC materials grades.

PVC Grade Material	Mean Square Error (MSE)
KI-16H	2.705474
KI-AV10	1.484374
KI-26H	2.037342
KI-AV2	0.855364
ASTM 667-98	2.705474
ASTM 668-98	1.278916

From the results, it can be seen that the artificial neural network is capable of predicting the extrusion process parameters for any grade of PVC thermoplastic that is used in cable manufacturing industries. Only PVC thermoplastic material was considered in this thesis due to the unavailability of real manufacturing data for different grades of PE thermoplastic materials. The training, validation, and testing MSE recorded in this study are $3.85525\;\times 10^{-10}$, $3.85525\;\times 10^{-6},$ and $8.496995\;\times 10^{-6}$ respectively. The regression values were observed to be 0.99364, 0.99628, and 0.99185 for the training, validation, and testing dataset respectively. Six (6) different grades of PVC were used with the developed model to attempt to predict the process parameters and the results were satisfactory. The results of this work, when compared with the best work in literature so far, have proven to be better by providing a reduced MSE value [25]. reported eight different methods of predicting product quality with the best method having an MSE value of $9.845137448147556\;\times \;10^{-5}$ which is still higher than the MSE recorded in this thesis. The use of ANN in predicting extrusion process parameters can improve the thermoplastic extrusion process experience in cable manufacturing industries.

### 3.3 Prospects of using artificial neural network in the extrusion process

It can be observed from this study that the use of an artificial neural network can accurately predict the extrusion process parameters in thermoplastic extrusion. This can significantly improve the output quality and increase the production rate of electrical cables. Production managers in industries can be equipped with the appropriate tools which can enable them to produce quality cable insulation while eradicating the need to perform long experiments which can lead to waste of materials and increase the cost of production. The prospects of utilizing the artificial neural network in the extrusion process are endless as it can also be used for the control of the entire system. The neural network controller coupled with an extruder (which enables it to be able to predict future plant behaviors and select appropriate control input) which can optimize future performance. Figure 19 shows the block diagram of the proposed prediction neural network controller which can be utilized to predict future extruder plant behavior.

## 4 Conclusion

This study has examined the extrusion process in cable manufacturing industries and developed an artificial neural network model to predict extrusion process parameters. A multilayer perceptron neural network trained with backpropagation using the Levenberg Marquardt algorithm was developed for predicting extrusion process parameters. The artificial neural network model developed in this study was able to accurately predict the extrusion process parameters for different grades of PVC cable in the thermoplastic extrusion used cable manufacturing industries. The use of artificial neural networks can eradicate the need for trial and error techniques which can improve the output quality in the thermoplastic extrusion process, and it will also reduce production time and cost. An artificial neural network is best suited to solve an industrial problem because it can be applied to real manufacturing execution systems.

## Declarations

### Author contribution statement

Ayokunle Adesanya: Performed the experiments; Analyzed and interpreted the data; Wrote the paper.

Ademola Abdulkareem: Conceived and designed the experiments; Wrote the paper.

Lambe Mutalub Adesina: Contributed reagents, materials, analysis tools or data.

### Funding statement

This research did not receive any specific grant from funding agencies in the public, commercial, or not-for-profit sectors.

### Competing interest statement

The authors declare no conflict of interest.

### Additional information

No additional information is available for this paper.
